# Health inequities in medical crowdfunding: a systematic review

**DOI:** 10.1186/s12939-025-02543-x

**Published:** 2025-06-10

**Authors:** Yingying Cai, Syafila Kamarudin, Xiaoyu Jiang, Baiyu Zhou

**Affiliations:** 1 Department of Communication, Universiti Putra Malaysia, Serdang, Selangor Malaysia; 2 Institute for Social Science Studies, Universiti Putra Malaysia, Serdang, Selangor Malaysia

**Keywords:** Crowdfunding, Medical crowdfunding, Health equities, Systematic review, Social determinants of health, Socioeconomic factors

## Abstract

**Background:**

Medical crowdfunding has emerged as a popular strategy to offset healthcare expenses in contexts of limited insurance coverage. While often framed as a democratizing and accessible financial tool, growing evidence indicates that success is unevenly distributed, raising concerns about its role in exacerbating health inequities.

**Methods:**

A systematic review was conducted in accordance with PRISMA guidelines, drawing from PubMed, CINAHL, Embase, Web of Science, and Scopus. Of 1,462 screened records, 33 studies met the inclusion criteria. Guided by the PROGRESS framework, we extracted data on socioeconomic determinants of health disparities. An inductive content analysis was employed to identify how equity was assessed across studies.

**Results:**

We identified three key categories of metrics used to assess equity in medical crowdfunding: funding outcomes, campaign visibility, and donor participation. Across these domains, substantial disparities were observed. Campaigns in rural or economically disadvantaged areas tended to have lower success rates. Racial and ethnic inequities were consistently documented, with non-white individuals receiving fewer and smaller donations than white counterparts. Gender disparities were complex, especially in transgender-related campaigns. Socioeconomic status and educational attainment were significantly associated with outcomes, accompanied by differences in access to social capital and the ability to craft persuasive narratives. In regions with high medical debt or limited insurance coverage, more crowdfunding campaigns appeared, but with lower overall success. These inequities were shaped and reinforced by platform algorithms and design features that privileged users with preexisting advantages.

**Conclusions:**

Rather than serving as a corrective to healthcare access gaps, medical crowdfunding often reflects and reinforces structural inequities. These findings challenge its portrayal as an equitable financing solution and highlight the need for policy interventions to ensure fairer access to healthcare resources.

**Supplementary Information:**

The online version contains supplementary material available at 10.1186/s12939-025-02543-x.

## Introduction

Medical crowdfunding, defined as the solicitation of donations through online platforms to cover healthcare expenses, has become an increasingly visible feature of healthcare financing across various national contexts. Platforms such as GoFundMe and Tencent Charity have facilitated substantial amounts of medical fundraising, positioning crowdfunding as a supplementary mechanism for addressing medical financial shortfalls [1, 2]. In the United States, medical campaigns constitute more than one third of all GoFundMe initiatives, making them the platform’s single largest category of donation-based crowdfunding efforts [3].

The rise of medical crowdfunding has occurred in tandem with broader structural shifts in healthcare financing, including the retreat of formal safety net institutions, the increasing financialization of healthcare, and rising out-of-pocket costs that limit access to essential services [4]. In systems characterized by limited insurance coverage and high treatment costs, crowdfunding provides an alternative channel for patients to seek financial support from personal networks and the general public [5]. While this approach may offer temporary relief in specific cases, its broader implications for health equity require further scrutiny.

Crowdfunding has become recognized as an empowering solution that enables numerous individuals or organizations that might not otherwise have access to funding to finance their projects [6]. However, this optimistic framing merits critical examination. Emerging empirical studies indicate that medical crowdfunding does not operate uniformly across populations and may, in some cases, mirror or even exacerbate existing social disparities [7, 8]. For instance, successful campaigns often rely on digital literacy, expansive social networks, and compelling personal narratives—resources that are not equally distributed across socioeconomic groups.

As crowdfunding becomes more integrated into the healthcare financing landscape, it is important to assess its implications through an equity lens. Kenworthy [7] proposed that medical crowdfunding should be recognized as a complex determinant of health, operating through technological (algorithms and platform politics), commercial (corporate influence and data privatization), and political channels (shifting norms around healthcare rights) that can exacerbate health inequities while appearing to address individual healthcare needs. Similarly, Berliner and Kenworthy [2] highlighted how donation platforms construct systems of perceived “worthiness,” wherein campaign success depends on factors such as narrative appeal, social visibility, and alignment with dominant norms. Building on these conceptual insights, empirical research has identified disparities in crowdfunding outcomes across various dimensions, including race [9, 10], gender [11, 12], socioeconomic status [13, 14], geographic location [15, 16], and health condition [3, 17].

While the body of research on medical crowdfunding has grown substantially in recent years, the field lacks a synthesized understanding of how disparities operate across multiple dimensions of identity and social position. Recent systematic reviews have focused primarily on success factors in medical crowdfunding [18] or its use for specific medical conditions [19], but a comprehensive synthesis of evidence regarding equity dimensions remains absent from the literature.

This systematic review aims to address this gap by examining the complex interplay between medical crowdfunding and health disparities. Following the definition of health equity as the absence of unfair and avoidable or remediable differences in health among population groups defined socially, economically, demographically or geographically [20], we synthesize evidence on how social determinants of health manifest in crowdfunding environments. Specifically, we address three primary research questions:

1. What metrics are used to evaluate medical crowdfunding campaigns and assess disparities in outcomes?
2. How do health disparities manifest across PROGRESS dimensions (Place of residence, Race/ethnicity, Occupation, Gender, Religion, Education, Socioeconomic status, and Social capital) in medical crowdfunding outcomes?
3. What factors mediate or exacerbate these disparities in crowdfunding contexts?

By systematically reviewing and synthesizing findings across multiple studies, geographic contexts, and methodological approaches, this review provides a comprehensive analysis of the equity implications of medical crowdfunding and the metrics used to assess campaign outcomes. The findings offer important insights for healthcare providers, policymakers, and platform designers navigating increasingly financialized healthcare landscapes.

## Methods

### Article identification and selection

To ensure comprehensive coverage, a systematic search strategy was employed, utilizing PubMed, CINAHL, Embase, Web of Science, and Scopus as the primary databases for literature retrieval. The search was conducted on October 15, 2024, without time restrictions and was limited to articles written in English. The following search terms were used: (crowdfund* OR “crowd fund*” OR “peer-to-peer fund*” OR “online fund*”) AND (health* OR medic* OR disease* OR illness* OR hospital* OR treatment* OR “medical bill*” OR “healthcare cost*”). This search yielded 460 records from PubMed, 100 from CINAHL, 180 from Embase, 396 from Web of Science, and 323 from Scopus, for a total of 1,459 articles. An additional 3 records were identified through other sources.

### Inclusion/ exclusion criteria

The identification and selection of articles followed the PRISMA guideline [21], which provides a standardized framework for transparent and systematic reporting. To ensure methodological rigor, two independent reviewers screened all titles and abstracts. The PROGRESS framework was applied during the data extraction and analysis phases as an organizing framework to systematically identify and categorize socioeconomic determinants of health inequalities in the selected studies. As defined by the PROGRESS framework, this approach captures a range of social determinants, including residence, race/ethnicity, occupation, gender, religion, education, socioeconomic status, and social capital [22].

**Fig. 1:**
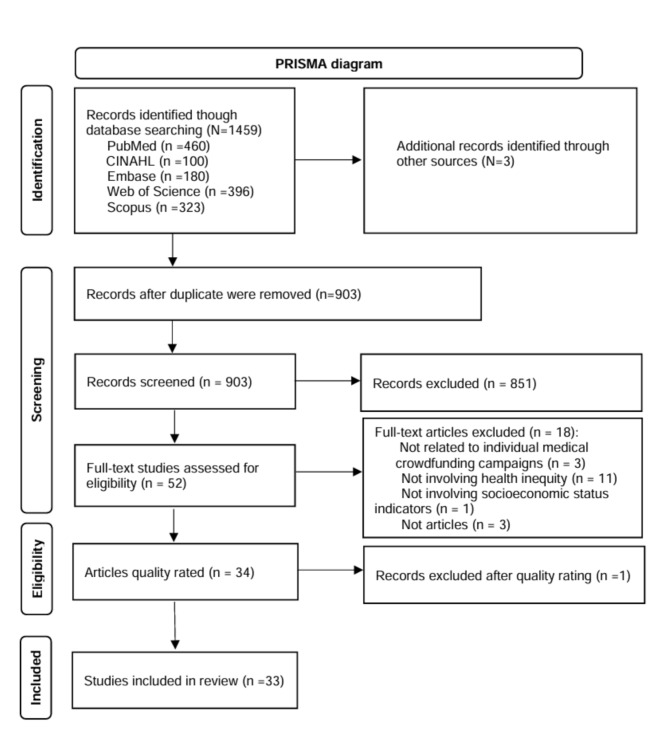
Article selection process

Figure 1 depicts the flow of publications through the identification, screening, eligibility evaluation, and inclusion stages, as well as the reasons for exclusions at each step. The inclusion criteria for studies were: (i) analysis of medical crowdfunding campaigns initiated by individuals; (ii) inclusion of socioeconomic status indicators; and (iii) examination of disparities in health outcomes. The exclusion criteria were: (i) studies focusing on organizational or charitable crowdfunding rather than individual campaigns, as these operate under different dynamics and motivations; (ii) studies that did not examine health inequalities as a primary or secondary outcome, as they would not address our research questions; (iii) studies lacking socioeconomic indicators necessary for analyzing disparities; and (iv) non-research articles such as commentaries, editorials, or opinion pieces that did not present original data or analysis. Any disagreements between reviewers during the screening process were resolved through discussion until consensus was reached, with a third reviewer consulted when necessary.

After removing duplicates, 903 unique records were retained. These records were screened for relevance, leading to the exclusion of 851 documents. The remaining 52 full-text articles were assessed for eligibility, and 18 were excluded for the following reasons: not related to individual medical crowdfunding campaigns (3), not involving health inequality (11), not involving socioeconomic status indicators (1), or not being research articles (3).

### Quality assessment

The quality appraisal stage was conducted to ensure that the selected studies adhered to rigorous methodological standards and provided reliable findings. For this purpose, the Mixed Methods Appraisal Tool (MMAT) by Hong, et al. [23] was employed. The MMAT is specifically designed to evaluate a wide range of study designs, including qualitative research, quantitative descriptive studies, randomized controlled trials, non-randomized studies, and mixed-methods research, offering a systematic approach to assess methodological soundness across diverse designs.

Each study was appraised using MMAT by two independent reviewers based on five predefined criteria relevant to the study design. Discrepancies were resolved through consultation with a third reviewer. Responses were categorized as “yes,” “no,” or “can’t tell.” Studies meeting at least four of the five criteria were included. Of the 34 studies assessed, one was excluded. A total of 33 studies met the quality criteria and were included in the final review.

### Analysis techniques

A structured data extraction process preceded the thematic analysis. A standardized extraction form was used to capture bibliographic information, research design, country of origin, crowdfunding platforms, socioeconomic indicators, campaign metrics, and key findings related to health inequalities. Two reviewers independently extracted data from each study, resolving discrepancies through discussion until consensus. The extracted data were organized in a matrix format to facilitate cross-study comparison.

We then conducted a systematic content analysis to synthesize findings across studies. First, appropriate units of analysis (e.g., words, phrases, meanings) were identified based on the research objectives [24]. Reviewers thoroughly read the materials and developed an initial coding framework, grouping abstracted information by similarity in meaning or recurring terminology. Subcategories were established and organized into overarching categories.

Using this framework, extracted data were categorized into thematic domains aligned with our research focus: (1) crowdfunding campaign metrics for equity assessment; (2) structural inequities in medical crowdfunding, including geographic, racial/ethnic, occupational, gender, religious, educational, and socioeconomic dimensions; and (3) expanded dimensions of inequity, such as narrative strategies, platform design mechanisms, and healthcare policy contexts. We applied inductive content analysis to each theme using both qualitative and quantitative data [25, 26], followed by a narrative synthesis to explore the complex relationship between crowdfunding and health inequalities.

## Result

### Characteristics of the analyzed literature

A total of 33 peer-reviewed studies were identified that examine medical crowdfunding across diverse geographic and platform contexts as shown in Table 1. The majority of these studies were conducted in the United States (*n* = 17), followed by China (*n* = 7), global or cross-national contexts (*n* = 6), Canada (*n* = 2), and India (*n* = 1). In terms of platform selection, GoFundMe.com emerged as the most frequently analyzed platform, appearing in 28 studies, either exclusively or in combination with others such as YouCaring and Watsi. Methodologically, quantitative research dominates the field (*n* =  25), with a smaller number of studies employing mixed methods (*n* = 6) or qualitative approaches (*n* = 2). This distribution reflects a prevailing focus on data-driven analyses while indicating a relative underrepresentation of qualitative insights.

**Table 1:** Summary of literature reviewed

Author	Country	Platform	Research Design
Barcelos [27]	Global	GoFundMe.com	Qualitative
Saleh, et al. [9]	Canada, UK, and USA	GoFundMe.com	Quantitative
Kenworthy, et al. [28]	USA	GoFundMe.com	Quantitative
Yang, et al. [29]	China	N/A	Quantitative
Podszus, et al. [15]	USA	GoFundMe.com	Quantitative
Silver, et al. [14]	USA	GoFundMe.com and InternetArchive.org	Quantitative
Shah [30]	India	Ketto	Quantitative
Faletsky, et al. [31]	USA	GoFundMe.com	Quantitative
Proelss, et al. [32]	Global	Watsi	Quantitative
Kimseylove, et al. [12]	USA	YouCaring and GoFundMe.	Mixed methods
Zhang, et al. [23]	China	Tencent Charity	Quantitative
Barcelos [11]	Global	GoFundMe.com	Quantitative
Mark, et al. [34]	Global	GoFundMe.com	Mixed methods
Barcelos and Budge [35]	Cross-national (6 countries)	GoFundMe.com	Quantitative
Zhang, et al. [10]	USA	GoFundMe.com	Quantitative
Waters, et al. [36]	USA	GoFundMe.com	Quantitative
Kenworthy [1]	USA	GoFundMe, YouCaring, Fundrazr and Watsi	Qualitative
Kenworthy and Igra [3]	USA	GoFundMe.com	Quantitative
Lee and Lehdonvirta [4]	USA	GoFundMe.com	Quantitative
Holler, et al. [37]	USA	GoFundMe.com	Quantitative
Berliner and Kenworthy [2]	USA	GoFundMe.com	Mixed methods
Davis, et al. [38]	Global	GoFundMe.com	Mixed methods
Zhu [39]	USA	GoFundMe.com	Mixed methods
van Duynhoven, et al. [16]	Canada	GoFundMe.com	Quantitative
Panjwani and Xiong [40]	USA	GoFundMe.com	Quantitative
Cheng and Ren [13]	China	N/A	Quantitative
Machado, et al. [41]	USA	GoFundMe、Fundly、YouCaring、GiveForward、MightyCause、Fundrazr and Indiegogo	Quantitative
Zheng, et al. [42]	China	Qschou.com	Quantitative
Ba, et al. [43]	China	Tencent Charity	Mixed methods
Wu and Peng [44]	China	Tencent Charity	Quantitative
Lai, et al. [17]	USA	GoFundMe.com	Quantitative
Zheng and Jiang [45]	China	Waterdrop Platform	Quantitative
Mark, et al. [46]	Global	GoFundMe.com	Quantitative

Note: “N/A” indicates that the original study did not explicitly mention the name of the crowdfunding platform used

### Crowdfunding campaign metrics for equity assessment

Table 2 summarizes the core metrics used across the reviewed studies to assess equity in medical crowdfunding campaigns. These metrics are organized into four categories: funding outcome metrics, campaign visibility metrics, donor participation metrics, and other structural indicators. Funding outcome metrics, such as “Amount Raised” and “Goal Amount,” are the most frequently employed, appearing in 29 and 26 studies respectively. Visibility metrics primarily include “Social Media Shares” and "Platform Engagement", though the latter are less commonly used. Donor participation is often measured by the number of donors (22 studies), with fewer studies examining average or maximum contributions. Other indicators, including campaign frequency and duration, are the least studied, with each appearing in fewer than five studies.

**Table 2:** Core metrics for assessing equity in medical crowdfunding campaigns

Category	Metric	Description	References
1. Funding Outcome Metrics	Goal Amount (26)	Target funding amount set by campaign organizers	[11]; [2]; [27]; [28]; [29]; [30]; [15]; [31, 32]; [17]; [33]; [34]; [9]; [14]; [35]; [12]; [36]; [37]; [1]; [3]; [4]; [38]; [39]; [13]; [40]; [41]
	Amount Raised (29)	Actual funds collected through the campaign	[36]; [12]; [11]; [14]; [37]; [3]; [42]; [39]; [27]; [43]; [44]; [31]; [28]; [9]; [29]; [15]; [45]; [35]; [30]; [1]; [4]; [33]; [2]; [38]; [13]; [40]; [32]; [17]; [41]
	Percentage of Goal Achieved (17)	Proportion of goal amount successfully raised	[11]; [28]; [29]; [45]; [39]; [31]; [16]; [34]; [14]; [12]; [27]; [36]; [3]; [2]; [40]; [32]; [17]
	Success Rate (Binary) (7)	Whether campaign achieved defined success threshold	[45]; [10]; [15]; [9]; [35]; [30]; [39]
	Funding time (1)	The time taken to complete funding	[46]
	Campaigns with $0 raised (1)	The proportion of campaigns that didn’t raise any money	[3]
	Number of Successful Campaigns (1)	The count of campaigns that reached their funding goals	[4]
2. Campaign Visibility Metrics	Social Media Shares (18)	Number of times a campaign is shared on platforms	[12]; [38]; [2]; [17]; [42]; [14]; [39]; [43]; [44]; [29]; [35]; [36]; [1]; [4]; [33]; [13]; [40]; [41]
	Platform Engagement (4)	“Hearts,” likes, comments on campaign page	[2]; [9]; [29]; [1];
3. Donor Participation Metrics	Number of Donors (22)	Total individuals contributing to a campaign	[9]; [37]; [11]; [2]; [13]; [42]; [44]; [31]; [29]; [43]; [15]; [14]; [45]; [35]; [27]; [30]; [36]; [10]; [3]; [33]; [32]; [41]
	Average Donation Amount (9)	Mean value of individual contributions	[2]; [45]; [40]; [10]; [29]; [42]; [39]; [29]; [44];
	Largest Single Donation (1)	Highest individual contribution to a campaign	[29]
4. Others	Campaign Frequency (3)	The number of crowdfunding activities or campaigns	[16]; [4]; [17]
	Campaign duration (5)	The length of time a crowdfunding campaign runs	[12]; [36]; [38]; [31]; [32]

### Structural inequities in medical crowdfunding

This section presents systematic findings that demonstrate how medical crowdfunding outcomes reflect and potentially amplify social inequities across multiple dimensions.

#### Geographic disparities

Geographic location influence crowdfunding performance across multiple levels, including international, regional, and urban–rural scales. At the international level, international campaigns were associated with raising less money than US-based campaigns in the initial regression model without social media variables [36]. Within the United States, campaigns from states without Medicaid expansion experience lower success rates despite higher need [12], occurring more frequently in areas characterized by higher medical debt and uninsurance rates[3].

The urban-rural divide represents another geographic dimension of inequality. Ba, et al. [31] found that urbanicity strongly predicts campaign success in China, with rural fundraisers facing significant disadvantages in both donation amounts and donor counts. Research shows that medical crowdfunding campaigns are disproportionately concentrated in regions with higher socioeconomic status, with van Duynhoven, et al.'s [16] finding that 65.49% of Canadian cancer-related campaigns originated from middle-to-high income areas, suggesting that existing socioeconomic inequalities may be reproduced through crowdfunding practices.

Podszus, et al. [15] identified striking regional disparities within the United States as well, with campaigns from the Northeast and Midwest demonstrating significantly higher success rates compared to those from the South. Geographic variations in crowdfunding outcomes were observed even after accounting for other demographic factors, indicating that place of residence contribute to disparities in campaign success.

#### Race/ethnicity/cultural/linguistic factors

A growing body of research has identified racial and ethnic disparities in medical crowdfunding outcomes. Several studies report that campaigns supporting minoritized racial groups tend to raise less funding and have reduced success rates compared to those supporting white recipients. Saleh, et al. [9] found that Black fundraisers raised 11.5% less than their non-Black counterparts (95% CI: −19.0% to − 3.2%; *P* = 0.006), while being significantly underrepresented in crowdfunding platforms relative to their population share.

Similar racial disparities were documented by Kenworthy, et al. [29], who reported that Black recipients received approximately $22 less per donation on average (*p* = 0.03) and attracted fewer donations overall. Zhang, et al. [10] further corroborated these findings, demonstrating that white fundraisers received 17.68% higher average donations than Black fundraisers. Machado, et al. [40] found white organizers raised a median amount of $3,050 compared to $2,343 for Black organizers, reflecting a 30.2% higher median donation amount for White organizers.

The racial gap extends beyond Black-white disparities. Davis, et al. [44] revealed that Asian men and other persons of color received more numerous but smaller donations than white men, resulting in lower overall funding. For Hispanic beneficiaries, racial disparities were also evident, with Podszus, et al.'s [15] finding that campaigns for Hispanic children were significantly less likely to succeed than those for white children.

These disparities persist even when controlling for campaign features. Panjwani and Xiong [39] demonstrated that campaigns organized by individuals with distinctively African-American or Hispanic names were less successful than campaigns by organizers with white-sounding names, though the researcher identified differences in social networks as a possible explanation for these racial disparities rather than explicitly attributing them to racial bias in the crowdfunding environment.

#### Occupational determinants

Occupational status represents another dimension of inequality in medical crowdfunding, though it has received comparatively less attention in the literature. Zhang, et al. [27] found that a patient’s occupation significantly impacts medical crowdfunding success, with fundraising success rates decreasing sequentially when the patient’s occupation was national civil servant, professional skill worker, clerk, business and service worker, soldier, child, student, and public-spirited person.

Military affiliation, however, presents a notable exception to this pattern. Podszus, et al. [15] documented that military affiliation significantly boosted campaign success. These contrasting occupational findings may highlight how perceptions of deservingness are entangled with judgments about recipients’ social roles and contributions, potentially creating disadvantages for individuals in less visible or less culturally valorized occupations.

#### Gender-based disparities

Gender-based differences have been observed in crowdfunding outcomes. Saleh, et al. [9] reported that female individuals raised 5.9% less than male counterparts and were underrepresented among US campaigns. Kenworthy, et al. [29] similarly found that female recipients received fewer donations than males.

In the context of transgender healthcare specifically, gender-based disparities are pronounced. Kimseylove, et al. [12] observed that transgender men significantly outperformed transgender women in funding outcomes, with transgender women achieving only a 5.9% success rate compared to 17% for transgender men. Barcelos [11] documented that 67% of transgender medical crowdfunding campaigns were initiated by transgender men, while only 28% were by transgender women, despite transgender women facing higher costs for gender-affirming procedures. These disparities highlight what Barcelos [11] described as “transmisogyny” operating in crowdfunding contexts. Additionally, Kenworthy, et al. [29] found that women performed 78.4% of crowdfunding organizational labor, especially when fundraising for others, suggesting gender disparities in the distribution of “digital care work.”

Notably, Yang, et al. [43] observed that gender disparities common in other contexts were not significant in their study of Chinese medical crowdfunding on WeChat, noting that men and women do not differ significantly in their returns to social capital in this specific platform context. This suggests that platform-specific features and cultural contexts may mediate gender disparities in crowdfunding outcomes.

#### Religious affiliation influences

Although less extensively studied than other factors, timing related to religious holidays demonstrates significant effects on crowdfunding performance. Proelss, et al. [46] found that campaigns begun around religious holidays are funded more quickly than those posted during regular periods, with Christian holidays showing the highest funding speed compared to federal holidays or weekends. The researchers attribute this to a “warm-glow” effect, where religious observances may prompt donors to reflect more on their behavior and think more about others. This effect is especially visible during the religious holiday season, when donors appear to have an increased awareness of others’ needs, which induces more reflection on their own behavior and situation. The researchers found that religious holidays, federal holidays, and weekends are all statistically significantly related to higher funding speed, but the effect is notably stronger during religious holidays than on federal holidays or weekends. The study ruled out alternative explanations like tax benefits or simply having more leisure time during holidays, confirming that the “warm-glow” effect during religious observances appears to be the primary mechanism driving this timing advantage.

#### Educational attainment differences

Educational attainment has emerged as a significant predictor of crowdfunding success according to recent research. Zheng, et al. [42] found that patients with college education raised 88.1% more funds than those without college education in China. This educational advantage operated through specific mechanisms: college-educated patients had more detailed narratives and more school-related social connections who were willing to verify and distribute the campaigns, ultimately attracting more supporters and expanding the campaigns’ reach.

While college-educated beneficiaries demonstrated advantages across multiple performance metrics (more supporters, more frequent sharing, and more school-related verifiers), the research revealed an interesting trade-off. College education significantly increased the total number of supporters but did not lead to more generous individual donations. In fact, college-educated patients attracted “more but less generous supporters”—their broader network reach was counterbalanced by lower median donation amounts per supporter. These findings quantify the “social support returns” to college education specifically in the context of medical crowdfunding in China.

#### Socioeconomic status implications

Socioeconomic status (SES) has been identified as a significant factor associated with variations in crowdfunding outcomes, potentially operating through multiple mechanisms. Cheng and Ren [13] documented a clear SES gradient in crowdfunding performance, where each standard deviation increase in SES was associated with a 31.1% increase in funds raised and a 31.9% increase in goal completion rate. Higher-SES individuals also received 22.8% more donations and 17.1% more shares than lower-SES counterparts.

Silver, et al. [14] revealed how neighborhood deprivation influenced cancer crowdfunding outcomes, with campaigns from the most affluent neighborhoods raising $7,402.94 on average, compared to just $4,857.87 from the most deprived areas—a 26.07% disadvantage. This SES gradient persisted across multiple studies and contexts, creating a paradoxical situation where those with the greatest financial need often receive the least crowdfunding support.

Kenworthy [1] described this phenomenon as crowdfunding functioning like a “grinding stone” that perpetuates and potentially amplifies existing socioeconomic inequalities. Lee and Lehdonvirta [4] similarly found that medical crowdfunding resembled “friendfunding” more than a true safety net, with those possessing greater social and economic resources achieving consistently better outcomes.

#### Social capital and resource access

Social capital, defined as the resources embedded in social networks, has been identified in several studies as an important factor influencing crowdfunding success. Yang, et al. [43] found that kinship ties had the strongest positive effect on crowdfunding performance, followed by pseudo-kinship ties (friends, colleagues, alumni), with neighborhood and other weak ties showing minimal impact.

The importance of social capital manifests in metrics such as Facebook shares, which consistently predict higher fundraising amounts. Barcelos and Budge [36] demonstrated that Facebook sharing frequency was more predictive of crowdfunding success than the total number of friends, highlighting the importance of active network mobilization rather than mere network size.

Despite the established correlation between social media engagement and fundraising success demonstrated in previous studies, Zheng and Jiang's [41] research reveals a counterintuitive pattern where campaigns with higher sharing frequency actually achieved lower completion ratios, challenging conventional assumptions about network dissemination benefits. Their findings suggest sharing and monetary donation may represent alternative rather than complementary behaviors for audience members.

### Mechanisms reinforcing inequity in crowdfunding

Beyond demographic and socioeconomic factors, crowdfunding inequalities manifest through additional mechanisms that warrant focused attention.

#### Narrative strategies and perceived deservingness

Narrative strategies have been shown to affect crowdfunding outcomes, potentially reflecting and reinforcing existing social hierarchies. Silver, et al. [14] found that campaigns that framed beneficiaries as brave (+ 15.40%), warm (+ 13.80%), or using militaristic metaphors (+ 11.87%) raised significantly more money. However, these advantageous narrative features were used more frequently in campaigns from higher-SES counties, creating another pathway through which privilege translates into crowdfunding success.

These findings align with broader patterns of inequality observed across different identity dimensions in crowdfunding. Barcelos [28] identified how transgender crowdfunding campaigns relied on “normative transition narratives” that conformed to medical models and binary gender expectations. Campaigns that employed these narratives achieved greater success, but at the cost of reinforcing narrow conceptions of transgender identity and potentially marginalizing individuals with non-normative gender expressions.

Similarly, Mark, et al. [30] found that transplant patients’ skin cancer crowdfunding success varied significantly based on narrative elements: campaigns mentioning infectious complications, pancreas transplants, or patient death raised substantially more funds, while those featuring older patients raised significantly less. Exceptional campaigns were associated with narratives emphasizing disability from disease, reluctance to ask for help, or death, suggesting that certain presentations of suffering are perceived as more deserving of financial support than others, potentially creating disparities between fundraising success and actual medical necessity.

Similar patterns emerge across cultures. Zheng and Jiang [41] observed that medical crowdfunding campaigns employing optimistic narrative tones achieved higher success rates than those emphasizing financial burdens or moral obligations, indicating that emotional presentation styles significantly influence donation patterns. Their analysis of 50,000 fundraising campaigns on China’s Waterdrop Platform showed that optimistic expressions increased completion ratios, while financial burden narratives and moral mobilization had negative impacts.

#### Platform design mechanisms and their effects

Digital platform architecture and algorithmic mechanisms have been identified as potential contributors to disparities in crowdfunding outcomes. Kenworthy [1] documented how platform algorithms prioritize “trending” and initially successful campaigns, creating a visibility advantage that compounds early success. This creates a “rich get richer” dynamic where early donations generate algorithmic amplification, increasing visibility and attracting additional support.

Platform interfaces also encourage setting “achievable” targets to create the appearance of success, which can disadvantage those with greater financial needs. Kenworthy [1] described how platforms encourage low goals for “success optics,” particularly affecting marginalized users who may require larger amounts but feel pressured to set modest targets.

Furthermore, platforms typically showcase image-rich, frequently updated campaigns with strong early momentum, creating structural advantages for users with greater digital literacy, time resources, and initial social capital. These design choices, while ostensibly neutral, interact with existing social inequalities to produce disparate outcomes across demographic groups.

#### Healthcare policy environment contexts

The broader healthcare policy context has been associated with variations in crowdfunding patterns and outcomes. Kenworthy and Igra [3] found that while states with higher rates of medical debt and uninsurance tended to have more crowdfunding campaigns per capita, campaigns in those states raised significantly less money, indicating a misalignment between crowdfunding outcomes and systemic healthcare needs.

Faletsky, et al. [35] identified policy-driven disparities in transgender healthcare crowdfunding, with campaigns from states explicitly excluding gender-affirming care from Medicaid raising significantly less (average $998) than those from states with inclusive policies (average $3,371). Similarly, Kimseylove, et al. [12] found that transgender adolescents in states lacking Medicaid mandates for gender-affirming care were more likely to launch crowdfunding campaigns and generally raised smaller proportions of their fundraising goals, suggesting that restrictive policy environments are associated with greater reliance on—and lower returns from—crowdfunding.

Lai, et al. [17] observed similar patterns in fertility treatment crowdfunding, reporting that states without insurance mandates had higher campaign frequencies and tended to raise lower median amounts per campaign. These findings suggest that crowdfunding serves not as a remedy for healthcare policy gaps, but rather as a signal of persistent unmet financial needs, particularly among underserved populations.

The intersection of policy contexts with crowdfunding outcomes highlights the limitations of individualized crowdfunding as a response to structural healthcare access problems. Rather than compensating for policy deficiencies, crowdfunding often reproduces and potentially amplifies the very inequalities created by inadequate healthcare systems. inequalities are maintained or exacerbated in digital fundraising environments.

## Discussion

### Structural and digital drivers of health inequities

To clarify how these drivers operate, we identify four intersecting mechanisms through which medical crowdfunding reinforces health inequities: (1) the reinforcement of preexisting social disparities; (2) political-economic access gaps and structural exclusion; (3) narrative deservingness and cultural gatekeeping; and (4) digital stratification through platform logics and algorithmic visibility.

#### Reinforcement of health inequities

Our analysis reveals that medical crowdfunding functions less as a tool for mitigating health disparities and more as a mechanism that reproduces and intensifies existing inequities. Individuals who face structural barriers to healthcare access often have the greatest financial need but the least capacity to launch successful campaigns. These individuals typically lack the technological access, narrative fluency, and social visibility required to attract donor support and are subject to biased evaluations of their “worthiness” by potential contributors. These findings support Kenworthy's [7] conceptualization of crowdfunding as a technological, commercial, and political determinant of health, and extend this framework by providing empirical evidence that quantifies how these disparities manifest across diverse global contexts.

Furthermore, our results indicate that medical crowdfunding systematically privileges individuals with pre-existing stocks of social, economic, and cultural capital, while creating additional structural barriers for historically marginalized groups. Although not explicitly framed in these terms, this pattern is consistent with Bourdieu's [47] theory of capital conversion, which provides a compelling lens for interpreting our findings. Individuals with greater cultural capital (e.g., higher levels of education, narrative competence), social capital (e.g., extensive and influential networks), and symbolic capital (e.g., identities aligned with dominant social norms) are more likely to convert these intangible resources into tangible economic outcomes—namely, crowdfunding success. In contrast, those lacking such capital are systematically disadvantaged, reinforcing broader patterns of social exclusion within digital health economies.

#### Political economy and access gaps

Medical crowdfunding should be understood within broader neoliberal healthcare transformations that increasingly individualize responsibility for health. Crowdfunding shifts healthcare financing from collective risk-pooling mechanisms to individual responsibility and personal network mobilization. Geographic disparities demonstrate that crowdfunding functions not as a solution to systemic healthcare gaps but rather as an imperfect adaptation to policy failures. The significantly lower crowdfunding success rates observed in states without Medicaid expansion [12] and in those with exclusionary policies toward transgender healthcare [35] underscore that crowdfunding cannot meaningfully compensate for structural deficiencies in health insurance coverage.

Furthermore, crowdfunding platforms operate as profit-seeking entities whose commercial interests shape user experiences and outcomes. Platform architectures that prioritize “trending” campaigns, encourage modest goals for success metrics, and reward early momentum create structural advantages for users with greater digital literacy, time resources, and initial capital [1].

#### Narrative deservingness and moral judgments

Crowdfunding success is shaped not solely by the objective severity of medical need, but by perceptions of deservingness—judgments that are deeply informed by cultural narratives and social biases. The persistent racial disparities observed in crowdfunding outcomes suggest that racial bias operates within these digital platforms much as it does in offline health and economic systems. Moreover, the documented fundraising advantages associated with specific narrative strategies [14]reveal how crowdfunding imposes additional labor demands on patients: namely, the work of crafting emotionally resonant, culturally legible appeals. As Berliner and Kenworthy argue [2]this dynamic constitutes a form of suffering-based economic redistribution, wherein access to healthcare becomes contingent upon the ability to perform suffering in ways that align with dominant moral expectations. Our findings corroborate this, demonstrating that campaigns invoking culturally valorized illness narratives—such as those emphasizing bravery or employing militaristic metaphors—and those aligning with normative gender representations, consistently receive greater financial support. This reinforces hierarchies of medical deservingness that may bear little relation to actual clinical urgency or need.

#### Platform logics and digital stratification

Our analysis positions digital crowdfunding platforms not as neutral intermediaries but as active sociotechnical agents that shape healthcare financing outcomes. As Kenworthy [1] demonstrates, platform algorithms, interface design features, and content promotion mechanisms collectively create structural advantages for users with greater technological access, digital literacy, and temporal flexibility. These mechanisms not only reinforce pre-existing social inequalities but also obscure them behind an affective discourse of empowerment and opportunity, further complicating public perceptions of fairness in medical fundraising.

The documented advantage of early donations creating momentum effects demonstrates how platform mechanics can amplify initial inequalities. Similarly, the advantage conferred by more frequent updates, image sharing, and social media promotion suggests that crowdfunding success depends not just on medical need but on sustained digital engagement—a resource-intensive requirement that disadvantages those with limited time, technological access, or digital literacy. These findings support the opinion that digital platforms actively shape health outcomes through their design choices, algorithmic decisions, and commercial imperatives [48]. These infrastructural platforms that increasingly mediate access to essential services like healthcare financing, making their inequality-producing effects particularly consequential [49].

### Implications for healthcare policy and practice

Our findings have significant implications for healthcare policy and clinical practice. First, they challenge the notion that crowdfunding represents a viable solution to healthcare financing gaps. The systematic disparities documented across multiple studies suggest that crowdfunding reproduces and potentially amplifies the very inequalities it purports to address. This indicates that more robust, equitable healthcare financing systems—not more crowdfunding—are needed to address healthcare access barriers.

Second, our findings highlight the need for healthcare providers to recognize the uneven landscape of crowdfunding success when considering financial options for patients. Clinicians should be aware that recommending crowdfunding as a financing strategy may be significantly more viable for some patients than others, potentially reinforcing disparities in care access. This suggests the importance of developing more equitable navigation resources and financial counseling approaches that account for these documented disparities.

Third, our analysis points to potential regulatory interventions in crowdfunding platforms themselves. The documented disparities suggest that platforms could implement equity-enhancing features such as promotion algorithms that counterbalance rather than amplify initial advantages, verification mechanisms that reduce bias in donor decision-making, or matching programs that direct additional support to campaigns from disadvantaged groups.

### Limitations and future research directions

While our review synthesizes evidence across multiple studies, several important limitations should be acknowledged. First, while traditional frameworks in health economics and the social determinants of health are extensively employed, relatively limited scholarly attention has been directed toward digital-era determinants—such as platform algorithms, digital literacy, and accessibility—which are increasingly recognized as influential in shaping the dynamics of medical crowdfunding. Including these factors could provide a more comprehensive understanding of the mechanisms driving disparities. Second, the study’s reliance on English-language databases introduces a potential selection bias, limiting its applicability to non-English-speaking and underrepresented regions. Expanding the scope to include studies from diverse linguistic and geographic contexts would ensure a broader and more inclusive perspective on health inequities in medical crowdfunding. Third, the rapid evolution of platform mechanics and healthcare financing landscapes means findings may evolve over time.

Future research should address several key gaps identified in our review. Future research should examine key gaps in medical crowdfunding, particularly how intersecting factors—such as race, gender, and age—shape disparities in donor behavior and campaign outcomes [29]. Age-related inequities also persist, with campaigns for older individuals consistently raising less than those for younger ones, even after controlling for other variables [50], reflecting broader societal perceptions of worthiness. In addition to demographic factors, platform-specific structures—such as algorithmic visibility and social network integration—may either exacerbate or mitigate these disparities [13]. Finally, further investigation into illness-related biases is needed, as campaigns for less severe but more prevalent conditions often perform better, indicating that cultural assumptions about deservingness influence digital giving patterns [31, 33].

## Conclusion

This systematic review demonstrates that medical crowdfunding operates not as an equality-enhancing alternative to traditional healthcare financing but rather as a mechanism that largely reproduces and potentially amplifies existing social hierarchies. The documented disparities across geographic, racial, socioeconomic, and other dimensions reveal crowdfunding transforms social advantage into healthcare access.

These findings contribute to our understanding of how digital platforms mediate health inequities in contemporary healthcare systems. Rather than democratizing healthcare financing, crowdfunding creates a system where personal networks, narrative skills, and socially valued identities determine healthcare access—reinforcing rather than challenging structural determinants of health.

As medical crowdfunding continues to grow as a healthcare financing mechanism, addressing these systematic disparities becomes increasingly urgent. Policymakers, healthcare providers, platform designers, and researchers need to work collaboratively to ensure that crowdfunding does not further entrench healthcare inequities in an era of growing healthcare financialization and individualization of responsibility.

## Electronic supplementary material

Below is the link to the electronic supplementary material.

Supplementary Material 1

## Data Availability

No datasets were generated or analysed during the current study.
